# Proteomic risk markers for coronary heart disease and stroke: validation and mediation of randomized trial hormone therapy effects on these diseases

**DOI:** 10.1186/gm517

**Published:** 2013-12-27

**Authors:** Ross L Prentice, Shanshan Zhao, Melissa Johnson, Aaron Aragaki, Judith Hsia, Rebecca D Jackson, Jacques E Rossouw, JoAnn E Manson, Samir M Hanash

**Affiliations:** 1 Division of Public Health Sciences, Fred Hutchinson Cancer Research Center, 1100 Fairview Ave N, P.O. Box 19024, Seattle, WA 98109, USA; 2 Research and Development, AstraZeneca LP, 1971 Rockland Road, Wilmington, DE 19803, USA; 3 Division of Endocrinology, The Ohio State University, 376 West Tenth Avenue, Suite 205, Columbus, OH 43210, USA; 4 WHI Project Office, National Heart, Lung, and Blood Institute, National Institutes of Health, 6701 Rockledge Drive, Bethesda, MD 20892, USA; 5 Division of Preventive Medicine, Brigham and Women’s Hospital, Harvard Medical School, 900 Commonwealth Avenue, Boston, MA 02215, USA; 6 Department of Clinical Cancer Prevention, Red and Charline McCombs Institute for the Early Detection and Treatment of Cancer, The University of Texas MD Anderson Cancer Center, 6767 Bertner Street, Houston, TX 77030, USA

## Abstract

**Background:**

We previously reported mass spectrometry-based proteomic discovery research to identify novel plasma proteins related to the risk of coronary heart disease (CHD) and stroke, and to identify proteins with concentrations affected by the use of postmenopausal hormone therapy. Here we report CHD and stroke risk validation studies for highly ranked proteins, and consider the extent to which protein concentration changes relate to disease risk or provide an explanation for hormone therapy effects on these outcomes.

**Methods:**

Five proteins potentially associated with CHD (beta-2 microglobulin (B2M), alpha-1-acid glycoprotein 1 (ORM1), thrombospondin-1(THBS1), complement factor D pre-protein (CFD), and insulin-like growth factor binding protein 1 (IGFBP1)) and five potentially associated with stroke (B2M, IGFBP2, IGFBP4, IGFBP6, and hemopexin (HPX)) had high discovery phase significance level ranking and an available ELISA assay, and were included in case-control validation studies within the Women’s Health Initiative (WHI) hormone therapy trials. Protein concentrations, at baseline and 1 year following randomization, were assessed for 358 CHD cases and 362 stroke cases, along with corresponding disease-free controls. Disease association, and mediation of estrogen-alone and estrogen plus progestin effects on CHD and stroke risk, were assessed using logistic regression.

**Results:**

B2M, THBS1, and CFD were confirmed (*P* <0.05) as novel CHD risk markers, and B2M, IGFBP2, and IGFBP4 were confirmed as novel stroke disease risk markers, while the assay for HPX proved to be unreliable. The change from baseline to 1 year in B2M was associated (*P* <0.05) with subsequent stroke risk, and trended similarly with subsequent CHD risk. Change from baseline to 1 year in IGFBP1 was also associated with CHD risk, and this change provided evidence of hormone therapy effect mediation.

**Conclusions:**

Plasma B2M is confirmed to be an informative risk marker for both CHD and stroke. The B2M increase experienced by women during the first year of hormone therapy trial participation conveys cardiovascular disease risk. The increase in IGFBP1 similarly conveys CHD risk, and the magnitude of the IGFBP1 increase following hormone therapy may be a mediator of hormone therapy effects. Plasma THBS1 and CFD are confirmed as CHD risk markers, and plasma IGFBP4 and IGFBP2 are confirmed as stroke risk markers.

**Clinical trials registration:**

ClinicalTrials.gov identifier: NCT00000611

## Background

Cardiovascular disease (CVD), particularly coronary heart disease and stroke, remains the leading cause of death in the United States among both women and men in all racial and ethnic groups. CHD is the designated cause for about 25% of all deaths and stroke for an additional 12% [1]. Risk factor epidemiology has played a crucial role in attempts to understand CVD mechanisms and pathways, and has led to the identification of effective approaches to disease prevention, for example through the treatment of hypertension [2], hypercholesterolemia [3], and arguably chronic inflammation [4]. Risk factor data, such as those arising from the Framingham Study cohort, have been effectively used to develop risk prediction models for CHD [5,6] and for stroke [7,8].

Both the age-incidence pattern and the strength of association for some risk factors differ between women and men, and it has been standard procedure to study CVD risk factors and risk prediction models in a sex-specific manner. Some studies have examined the ability of non-traditional risk factors to add to discrimination between CVD cases and controls. For example, an ischemic stroke study [9] showed that the estimated area under the receiver-operator-characteristic curve (AUC) among women in the Atherosclerosis Risk in Communities cohort increased from 0.83 to only 0.84 when certain non-traditional risk markers were included. Corresponding numbers were 0.76 and 0.80 among men. A study of CHD risk prediction models [10] in the Women’s Health Initiative (WHI) postmenopausal hormone therapy (HT) trial cohort found that the AUC increased from 0.73 to 0.75 when certain non-traditional risk factors were added. While these analyses imply an ability to assign CHD and stroke risk estimates that vary by several-fold among individuals, there is still a limited ability to identify individuals who are highly likely to develop disease, say, in the next 5 years. Additional blood-based biomarkers may lead to improvements in risk discrimination and risk prediction.

Blood biomarkers also have potential to provide biological insights into the effects of interventions on CVD outcomes. In particular, the pathways influenced and the key mediators of the effects of postmenopausal estrogen (E-alone) and estrogen plus progestin (E + P) on CVD remain substantially unknown. In the WHI randomized controlled trials these effects include an early elevation of CHD risk with E + P [11] that was less apparent for E-alone [12], and sustained elevations in stroke risk, of a similar magnitude with E + P [13] and E-alone [14].

We have carried out proteomic discovery work using an Intact Protein Analysis System [15] to compare, for about 370 proteins, pre-diagnostic plasma concentrations between CHD cases and matched controls, and between stroke cases and matched controls, drawn from the WHI Observational Study cohort [16]. We confirmed [17] the associations of beta-2-microglobulin (B2M) with short-term CHD risk, and the association of insulin-like growth factor-binding protein 4 (IGFBP4) with short-term stroke risk by comparing baseline blood concentrations for these proteins among women developing these diseases during the first year of participation in the WHI HT trials to 1-1 matched controls, using enzyme-linked immunosorbent assays (ELISAs). The association of these markers with longer-term CHD and stroke risk has yet to be studied. Importantly, there were several other proteins having empirical support for CHD or stroke risk association in our discovery research, with commercially available ELISAs. Here we report on these proteins collectively in relation to CHD and stroke incidence in the WHI HT trials, both as novel disease risk markers and as potential mediators of corresponding postmenopausal HT effects on CHD and stroke risk.

## Methods

### Study subjects and outcome ascertainment

Women who were postmenopausal and in the age range of 50-79 years enrolled in the WHI HT trials during 1993 to 1998, including 10,739 women who were post-hysterectomy in the E-alone trial and 16,608 women with uterus in the E + P trial. Of these, women who experienced CHD or stroke through February 2001 were included in a CVD biomarker case-control study [18,19]. Controls who were free of CVD through this date were matched 1-1 to cases on age, randomization date, hysterectomy status, and prevalent study disease at baseline in each trial. These cases and controls were well-characterized in terms of traditional risk factors. One hundred incident stroke cases arising during subsequent HT trial follow-up, and 1-1 matched controls using the same matching criteria, were subsequently added to enhance the ability to study the two diseases separately. A small number of the selected controls developed the disease of their matched case by the end of the planned trial intervention phase (8 April 2005) and are included in the case group here, giving a total of 358 CHD cases and a corresponding 352 controls, and a total of 362 stroke cases and a corresponding 346 matched controls for whom baseline plasma protein concentrations were assessed. Of these, 106 CHD cases and 68 stroke cases had their disease events during the first year following randomization. For all other cases and controls plasma protein concentrations were also assessed from blood drawn at 1 year following randomization.

CHD in the HT trials is defined as non-fatal myocardial infarction (MI) or death due to coronary heart disease. Disease event ascertainment involved physician adjudication based on the review of pertinent documents at each clinical center, and further adjudication by a central committee [20] with agreement rates of 90% for MI and 97% for death due to coronary heart disease, between local and central adjudication. Cases of hospitalized stroke were based on rapid neurologic deficit attributable to arterial obstruction or rupture, or a demonstrable lesion compatible with acute stroke [13]. Central neurologists reviewed all stroke cases, as well as transient ischemic attacks and self-reports of stroke. Strokes were classified as ischemic or hemorrhagic, as well as according to various outcome scales.

All participants provided written informed consent for their HT trial and their overall WHI participation. The related protocols were approved by the Institutional Review Board of the Fred Hutchinson Cancer Research Center and each of the 40 participating clinical centers. The research was conducted in accordance with the Helsinki Declaration and with pertinent local legislation.

### Specimen preparation and analysis

Fasting blood specimens were obtained at baseline in WHI as a part of eligibility screening and at 1 year following randomization, for clinical trial women. Serum and plasma were sent to a central laboratory and stored at -70°C. Plasma specimens for this project were plated so that case and matched control specimens were analyzed together. For cases occurring after the first year from randomization, the 1-year plasma specimens were plated with baseline specimens for concurrent ELISA analyses. Assays for each plasma sample followed ELISA kit manufacturer (R and D Systems, Minneapolis, MN, USA, for IGFBP1, IGFBP2, IGFBP4, IGFBP6, ORM1, THBS1, and CHD; CalBiotech, Spring Valley, CA, USA, for B2M; Abnova, Taipei, Taiwan, for HPX) recommendations. All samples were assayed with sample characteristics blinded, and in duplicate. Quality control activities included 5% blind duplicate analyses, using plasma from postmenopausal women outside of the HT trial cohorts. The reliability of the ELISA measurements was assessed by examining intra-class correlations between blind duplicates. Each protein concentration was reliably measured, with intra-class correlations ranging from 0.79 to 0.97, with the exception of HPX where the intra-class correlation was 0.38. Linear dilution curves were examined and a dilution was selected for each analyte that was above the detection threshold and below saturation. The dilutions applied were 1:50, 1:10,000, 1:1,500, 1:3,000, and 1:25 for B2M, ORM1, THSB1, CFD, and IGFBP1, respectively; and 1:250, 1:50, 1:500, and 1:400 for IGFBP2, IGFBP4, IGFBP6, and HPX, respectively.

### Proteomic biomarker selection

The in-depth proteomic discovery methodology [15,21] led to nine proteins in a false discovery rate (FDR) bin [22] of less than 20% for CHD and 11 such proteins for stroke [17]. Of these B2M, ORM1, THBS1, CFD, and IGFBP1 were selected on the basis of not being established as CHD risk markers, and having commercially available ELISA assays. The same criteria applied to the novel stroke candidates led to the selection of IGFBP2, IGFBP4, and IGFBP6, and HPX. B2M was also assessed for stroke cases and controls, based on its CHD association and a nominal *P* value of 0.03, even though FDR bin was higher (0.31) for this protein.

The CVD proteomic discovery work was complemented by additional IPAS analyses comparing blood protein concentrations at baseline and at 1 year following randomization for 50 women who adhered to active intervention during the first year of the E-alone trial [23], and 50 women who adhered to active intervention in the E + P trial [24]. These analyses suggested many proteomic changes following 1 year of use of these preparations, with 169 (44.7%) of the 378 proteins quantified having some evidence (nominal *P* <0.05) of change for one or both of E-alone or E + P [24]. Proteins with changed concentrations contributed to multiple biologic pathways relevant to the observed clinical effects of HT, including coagulation, inflammation, immune response, metabolism, cell adhesion, growth factors, and osteogenesis. The estimated 1-year *versus* baseline concentration ratios were very similar for E-alone and E + P for most highly ranked proteins, supporting the notion of combining proteomic analyses across the two trials.

Of the risk marker candidates selected here, B2M, CFD, and IGFBP1 for CHD, and each of the five proteins selected for stroke were among the proteins whose concentrations were observed to change (nominal *P* <0.05) as a result of HT [24].

### Statistical methods

Principal association analysis estimated CHD or stroke odds ratios (ORs) as a function of log-transformed baseline biomarker values using binary logistic regression of case (1) *versus* control (0) status [25]. For either disease, the logistic regression model also included systolic and diastolic blood pressure, cigarette smoking, diabetes, prior HT use, and body mass index, as well as the case-control matching factors (age, hysterectomy status, randomization year, prior history of study disease).

Analyses to examine the extent to which treatment-related changes in protein concentrations between baseline and 1 year following randomization can provide an explanation for E-alone and E + P effects on CHD and stroke, also relied on binary logistic regression. Analyses of the type just described, but based on cases occurring after the first year from HT trial enrollment and all controls for the specific trial, were carried out to estimate HT effects on disease OR and to examine the possibility of an OR dependence on the (log-transformed) baseline level of a biomarker under study. A biomarker mediation analysis then proceeded by adding the (log-transformed) year 1 biomarker value or equivalently, the logarithm of the year 1-to-baseline biomarker ratio, to the regression model, and examining the change in the HT OR following the year-1 biomarker addition.

## Results

Table 1 shows some characteristics of contributing cases and controls, separately for CHD and stroke, and separately for the E-alone and E + P trials. Compared to E + P trial women, E-alone trial women tended to have higher BMI, and to be more likely to have used postmenopausal hormones prior to trial enrollment.

**Table 1:** **Characteristics of CHD cases and controls and stroke cases and controls from the WHI postmenopausal HT trials of estrogen plus progestin (E + P) and estrogen-alone** (**E-alone): Mean ± SE for continuous baseline covariates, N** (**%) for categorical baseline covariates**

**CHD**						
	**E + P**			**E-alone**		
	**Cases**	**Controls**	***P *** **value**^**a**^	**Cases**	**Controls**	***P *** **value**^**a**^
N	206	202		152	150	
Age^b^ (years)	66.61 (7.55)	66.49 (7.44)	0.87	67.55 (6.01)	67.58 (6.03)	0.96
BMI (kg/m^2^)	28.81 (5.61)	27.57 (5.38)	0.02	30.21 (5.71)	29.49 (5.86)	0.28
Systolic BP (mmHg)	135.04 (18.62)	130.32 (17.85)	0.01	138.48 (18.50)	129.75 (17.77)	<.0001
Diastolic BP (mmHg)	77.04 (10.26)	75.39 (8.83)	0.08	76.29 (10.81)	74.56 (8.81)	0.13
Race (%)			0.77			0.66
White	185 (91.6)	180 (89.6)		117 (78.0)	118 (79.2)	
Black	11 (5.4)	13 (6.5)		26 (17.3)	27 (18.1)	
Other	3 (3.0)	8 (4.0)		7 (4.7)	4 (2.7)	
Smoking (%)			0.001			0.11
Never	92 (46.0)	114 (57.0)		70 (46.7)	79 (53.4)	
Past	68 (34.0)	72 (36.0)		55 (36.7)	56 (37.8)	
Current	40 (20.0)	14 (7.0)		25 (16.7)	13 (8.8)	
History of CHD^b^ (%)			1			1
Yes	19 (9.2)	19 (9.4)		21 (13.8)	21 (14.0)	
No	187 (90.8)	183 (90.6)		131 (86.2)	129 (86.0)	
Treated diabetes (%)			0.04			0.0003
Yes	29 (14.1)	15 (7.4)		39 (25.7)	14 (9.3)	
No	177 (85.9)	187 (92.6)		113 (74.3)	136 (90.7)	
Prior use of HT (%)			0.04			0.13
Never	152 (73.8)	152 (75.2)		96 (63.2)	97 (64.7)	
Past	48 (23.3)	39 (19.3)		48 (31.6)	37 (24.7)	
Current	6 (2.9)	11 (5.4)		8 (5.3)	16 (10.7)	
**Stroke**						
	**E + P**			**E-alone**		
	**Cases**	**Controls**	***P *** **value**^**a**^	**Cases**	**Controls**	***P *** **value**^**a**^
N	182	176		180	170	
Age^b^ (years)	67.90 (6.61)	67.86 (6.61)	0.96	67.56 (6.34)	67.43 (6.49)	0.85
BMI (kg/m^2^)	28.04 (4.94)	27.81 (6.26)	0.70	29.66 (5.42)	28.55 (5.39)	0.06
Systolic BP (mmHg)	137.82 (19.79)	128.24 (15.20)	<.0001	139.93 (19.34)	132.87 (17.51)	0.0004
Diastolic BP (mmHg)	76.73 (10.43)	73.93 (9.35)	0.008	77.99 (9.29)	76.45 (9.21)	0.12
Race (%)			0.68			0.98
White	156 (87.6)	156 (90.2)		134 (74.4)	126 (75.4)	
Black	14 (7.9)	12 (6.9)		30 (16.7)	27 (16.2)	
Other	8 (4.5)	5 (2.9)		16 (8.9)	14 (8.4)	
Smoking (%)			0.002			0.98
Never	77 (42.5)	102 (59.3)		92 (52.9)	60 (53.9)	
Past	74 (40.9)	58 (33.7)		68 (39.1)	64 (38.3)	
Current	30 (16.6)	12 (7.0)		14 (8.0)	13 (7.8)	
History of stroke^b^ (%)			1			1
Yes	5 (2.7)	4 (2.3)		9 (5.0)	9 (5.3)	
No	177 (97.3)	172 (97.7)		171 (95.0)	161 (94.7)	
Treated diabetes (%)			0.005			0.05
Yes	20 (11.0)	5 (2.8)		28 (15.6)	14 (8.2)	
No	162 (89.0)	171 (97.2)		152 (84.4)	156 (91.8)	
Prior use of HT (%)			0.50			0.40
Never	135 (74.2)	129 (73.3)		92 (51.1)	87 (51.2)	
Past	42 (23.1)	38 (21.6)		75 (41.7)	64 (37.6)	
Current	5 (2.7)	9 (5.1)		13 (7.2)	19 (11.2)	

^a^*P* value from chi-square test.

^b^Case-control matching variables.

BMI, body mass index; BP, blood pressure.

Table 2 shows geometric means and 95% confidence intervals (CIs) for each selected analyte at baseline, for cases and controls separately, along with *P* values for their comparison. *P* values for testing equality of case-control differences between the two trials are also shown, and no between-trial differences were suggested. Combined trial comparisons show CHD case-control differences (*P* <0.05) for B2M and CFD, and stroke case-control differences for B2M and IGFBP4.

**Table 2:** **Geometric mean and 95% CI for baseline and year-1 biomarker values and for the year 1 to baseline ratio** (**change) for cases and controls from the WHI postmenopausal HT trials of estrogen plus progestin** (**E + P) and estrogen-alone** (**E-alone)**

**CHD**								
	**E + P**			**E-alone**			**Difference between cohorts**^**b**^	**Overall case *vs. *** **control difference**^**c**^
	**Cases**	**Controls**	***P *** **value**^**a**^	**Cases**	**Controls**	***P *** **value**^**a**^		
N	206	202		152	150			
B2M (mg/mL)	N = 206, 141^d^	N = 202, 138^d^		N = 152, 110^d^	N = 150, 108^d^			
Baseline	0.13 (0.06, 0.29)	0.11 (0.06, 0.22)	<.001	0.13 (0.06, 0.32)	0.12 (0.05, 0.27)	0.05	0.44	0.0001
Year 1	0.15 (0.07, 0.31)	0.13 (0.06, 0.27)	0.01	0.15 (0.06, 0.38)	0.14 (0.07, 0.30)	0.04	0.87	0.003
Change	1.04 (0.51, 2.11)	1.03 (0.55, 1.94)	0.68	1.02 (0.48, 2.17)	1.00 (0.60, 1.67)	0.18	0.69	0.67
ORM1 (mg/mL)	N = 204, 139	N = 202, 138		N = 133, 96	N = 150, 108			
Baseline	0.52 (0.27, 1.02)	0.49 (0.27, 0.91)	0.10	0.52 (0.25, 1.08)	0.51 (0.25, 1.02)	0.61	0.52	0.16
Year 1	0.47 (0.24, 0.91)	0.46 (0.22, 0.94)	0.66	0.49 (0.25, 0.96)	0.47 (0.2, 1.08)	0.95	0.97	0.32
Change	0.93 (0.50, 1.71)	0.93 (0.48, 1.78)	0.85	0.93 (0.48, 1.79)	0.91 (0.51, 1.63)	0.41	0.68	0.75
THBS1 (ug/mL)	N = 172, 125	N = 175, 124		N = 145, 106	N = 108, 108			
Baseline	1.98 (0.18, 21.35)	2.10 (0.19, 23.82)	0.44	1.85 (0.24, 14.36)	2.36 (0.24, 23.08)	0.02	0.35	0.14
Year 1	1.98 (0.2, 19.12)	2.16 (0.19, 24.55)	0.27	2.16 (0.27, 17.14)	2.39 (0.25, 23)	0.10	0.95	0.38
Change	0.96 (0.12, 7.96)	0.94 (0.13, 6.67)	0.17	1.24 (0.16, 9.53)	0.97 (0.16, 5.76)	0.08	0.26	0.25
CFD (ug/mL)	N = 204, 139	N = 202, 138		N = 150, 108	N = 131, 91			
Baseline	0.79 (0.35, 1.74)	0.72 (0.37, 1.4)	0.003	0.78 (0.36, 1.67)	0.76 (0.4, 1.45)	0.22	0.27	0.02
Year 1	0.75 (0.32, 1.73)	0.7 (0.34, 1.42)	0.05	0.81 (0.33, 1.98)	0.77 (0.36, 1.65)	0.20	0.75	0.09
Change	0.98 (0.58, 1.65)	0.96 (0.65, 1.43)	0.26	1.04 (0.63, 1.70)	1.01 (0.72, 1.44)	0.22	0.95	0.30
IGFBP1 (ng/mL)	N = 201, 139	N = 201, 138		N = 150, 108	N = 145, 105			
Baseline	10.98 (1.56, 77.48)	10.71 (1.26, 90.8)	0.77	9.21 (1.09, 77.72)	7.61 (0.75, 76.76)	0.19	0.32	0.25
Year 1	15.70 (1.85, 133.37)	15.62 (1.81, 134.43)	0.85	17.67 (1.49, 209.42)	12.52 (0.91, 172.68)	0.06	0.12	0.16
Change	1.63 (0.33, 8.20)	1.40 (0.24, 8.15)	0.41	1.79 (0.37, 8.52)	1.87 (0.27, 12.83)	0.13	0.21	0.38
**Stroke**								
	**E + P**			**E-alone**			**Difference between cohorts**^**b**^	**Overall case *vs. *** **control difference**^**c**^
	**Cases**	**Controls**	***P *** **value**^**a**^	**Cases**	**Controls**	***P *** **value**^**a**^		
N	182	176		180	170			
B2M (mg/mL)	N = 181, 146^d^	N = 174, 146^d^		N = 179, 141^d^	N = 170, 134^d^			
Baseline	0.12 (0.04, 0.38)	0.11 (0.06, 0.20)	0.02	0.13 (0.06, 0.25)	0.11 (0.06, 0.22)	0.002	0.18	0.003
Year 1	0.12 (0.06, 0.26)	0.11 (0.06, 0.21)	0.03	0.13 (0.06, 0.27)	0.12 (0.06, 0.24)	0.06	0.84	0.001
Change	1.05 (0.33, 3.32)	0.99 (0.69, 1.40)	0.32	1.03 (0.64, 1.65)	1.04 (0.70, 1.56)	0.99	0.20	0.31
IGFBP2 (ng/mL)	N = 171, 136	N = 169, 139		N = 164, 128	N = 158, 125			
Baseline	80.9 (23.63, 276.99)	73.52 (15.27, 353.91)	0.53	72.55 (16.63, 316.48)	67.38 (15.28, 297.04)	0.36	0.85	0.14
Year 1	64.95 (16.88, 249.86)	66.11 (17.02, 256.78)	0.65	60.57 (12.3, 298.34)	57.99 (12.4, 271.3)	0.63	0.64	0.86
Change	0.85 (0.33, 2.17)	0.90 (0.40, 2.04)	0.42	0.86 (0.34, 2.17)	0.91 (0.32, 2.55)	0.15	0.96	0.16
IGFBP4 (ug/mL)	N = 175, 139	N = 170, 139		N = 168, 131	N = 164, 128			
Baseline	0.49 (0.22, 1.08)	0.45 (0.18, 1.09)	0.11	0.48 (0.21, 1.11)	0.43 (0.20, 0.95)	0.03	0.87	0.004
Year 1	0.56 (0.24, 1.35)	0.52 (0.23, 1.16)	0.05	0.57 (0.24, 1.39)	0.5 (0.21, 1.18)	0.01	0.47	0.003
Change	1.05 (0.54, 2.05)	1.05 (0.53, 2.10)	0.76	1.10 (0.59, 2.07)	1.03 (0.55, 1.93)	0.08	0.24	0.59
IGFBP6 (ug/mL)	N = 166, 130	N = 159, 133		N = 161, 126	N = 153, 118			
Baseline	0.15 (0.05, 0.43)	0.14 (0.05, 0.4)	0.56	0.15 (0.04, 0.47)	0.14 (0.04, 0.55)	0.99	0.89	0.55
Year 1	0.14 (0.05, 0.41)	0.13 (0.03, 0.51)	0.53	0.14 (0.04, 0.47)	0.14 (0.03, 0.59)	0.58	0.42	0.60
Change	0.92 (0.31, 2.75)	0.91 (0.25, 3.38)	0.99	0.96 (0.28, 3.31)	0.93 (0.33, 2.61)	0.50	0.86	0.57
HPX (mg/mL)	N = 175, 140	N = 170, 140		N = 169, 131	N = 164, 128			
Baseline	0.55 (0.33, 0.92)	0.54 (0.31, 0.95)	0.93	0.57 (0.31, 1.06)	0.54 (0.31, 0.97)	0.17	0.42	0.19
Year 1	0.52 (0.33, 0.82)	0.52 (0.31, 0.87)	0.95	0.54 (0.32, 0.9)	0.53 (0.33, 0.83)	0.35	0.78	0.42
Change	1.00 (0.64, 1.58)	0.99 (0.64, 1.51)	0.50	1.02 (0.67, 1.55)	1.04 (0.71, 1.52)	0.71	0.38	0.09

^a^*P* value from t-test for log-transformed biomarker values.

^b^For the *P* value between cohorts, a linear regression of log-biomarker value on trial, case *versus* control status and their interaction was fitted. The reported *P* value is the *P* value of the interaction.

^c^For the *P* value of overall difference between cases and controls, a linear regression of log-biomarker value on trial and case *versus* control status was fitted. The reported *P* value is the *P* value of the case *versus* control variable.

^d^Number of women at baseline and 1 year, respectively.

Case-control comparisons based on blood drawn at 1 year following randomization (year 1) are also shown in Table 2, excluding cases occurring in the first year of trial participation. For B2M, combined trial case-control differences at 1 year were evident for both CHD and stroke; and for IGFBP4, were evident for stroke.

More refined baseline analyte comparisons, using logistic regression, are shown in Table 3. These use all cases and controls for each disease, include indicator variables for treatment (active *vs.* placebo) and trial (E + P *vs*. E-alone), all control matching variables, and several other CVD risk factors (listed above). The ORs shown are for a 30% increment in the proteomic marker, a value well within the observed range of values for the biomarkers studied. These logistic regression analyses demonstrate positive associations of B2M and CFD, a modest inverse association of THBS1, and a possible positive association of IGFBP1 with CHD risk, each of which concurs in direction with the preceding proteomic discovery results. These analyses also imply positive associations of stroke risk with IGFBP2, IGFBP4, and B2M. Disease risk did not differ between the two trial cohorts, after controlling for the listed factors.

**Table 3:** **ORs** (**95% CIs) for a 30% baseline biomarker increment from analyses that include data from both WHI postmenopausal HT trials**

**CHD**					
	**B2M**	**ORM1**	**THBS1**	**CFD**	**IGFBP1**
Baseline biomarker	1.21 (1.09, 1.35)^a,c^	1.01 (0.89, 1.14)	0.97 (0.93, 1.00)	1.16 (1.03, 1.31)^c^	1.03 (0.99, 1.08)
Treatment^b^	1.32 (0.97, 1.81)	1.33 (0.97, 1.81)	1.34 (0.96, 1.87)	1.31 (0.95, 1.78)	1.29 (0.94, 1.77)
Trial^b^	1.13 (0.82, 1.56)	1.09 (0.79, 1.51)	1.02 (0.72, 1.44)	1.09 (0.79, 1.50)	1.05 (0.76, 1.47)
**Stroke**					
	**B2M**	**IGFBP2**	**IGFBP4**	**IGFBP6**	**HPX**
Baseline biomarker	1.18 (1.04, 1.34)^a,c^	1.10 (1.03, 1.18)^c^	1.16 (1.04, 1.28)^c^	1.01 (0.94, 1.09)	1.10 (0.95, 1.27)
Treatment^b^	1.90 (1.38, 2.62)	1.77 (1.27, 2.46)	1.76 (1.21, 2.54)	1.70 (1.22, 2.38)	1.79 (1.30, 2.48)
Trial^b^	1.09 (0.79, 1.50)	1.10 (0.79, 1.53)	1.09 (0.75, 1.59)	1.11 (0.79, 1.55)	1.13 (0.81, 1.56)

^a^All analyses also include baseline age, prior history of study disease, systolic and diastolic blood pressure, smoking history, treated diabetes history, prior HT use, and body mass index as regression variables to control confounding.

^b^Treatment, 1 - active; 0 - placebo; Trial, 1 - E + P trial (no hysterectomy), 0 - E-alone trial (post-hysterectomy).

^c^Significant at *P* = 0.05.

We previously reported [17] positive associations of baseline B2M with CHD risk and baseline IGFBP4 with stroke risk during the first year from randomization in the WHI HT trials. With the longer-term data analyzed here the estimated CHD OR (95% CI) for a 30% increment in baseline B2M is 1.28 (1.08, 2.54) following year 1, and the stroke OR (95% CI) for a 30% increment in baseline IGFBP4 is 1.16 (1.03, 1.30) following year 1. These are similar to the ORs for the first year [17], and for the overall time period as shown in Table 3.

Additional analyses (not shown) examined ORs for a 30% increase in baseline biomarker, separately in the placebo and treatment groups for each clinical trial.

The B2M associations with risk were evident in both placebo and treatment groups for both diseases. The CFD associations with CHD were primarily evident in the active treatment groups in both trials; whereas the IGFBP4 association with stroke was most evident in the placebo groups for both trials.

There were some noteworthy correlations among the protein concentrations considered for each disease. For CHD, baseline and year 1 B2M concentrations correlated positively with corresponding CFD concentrations in both the placebo and active hormone groups, in both the E-alone and E + P trials. For stroke, positive correlations of B2M with each of IGFBP2, IGFBP4, and IGFBP6 were also evident at baseline and year 1 in both treatment groups, and both trials. Table 4 shows ORs for the five analytes jointly, for each disease, in analyses that otherwise include the same regression variables as Table 3. The strongest associations in these analyses are for B2M and CHD and for IGFBP4 and stroke, while an inverse association of CHD risk with THBS1 and a positive association of stroke risk with IGFBP2 are also observed.

**Table 4:** **ORs** (**95% CIs) for a 30% baseline biomarker increment, from analyses that combine trials and treatment arms in the WHI postmenopausal HT trials, with biomarkers simultaneously modeled**

**Baseline biomarker**	**CHD**^**a**^	**Baseline biomarker**	**Stroke**^**a**^
B2M	1.19 (1.05, 1.35)^b,c^	B2M	1.11 (0.96, 1.29)^b^
ORM1	1.06 (0.93, 1.22)	IGFBP2	1.08 (1.00, 1.17)^c^
THBS1	0.96 (0.92, 0.99)^c^	IGFBP4	1.17 (1.04, 1.32)^c^
CFD	1.09 (0.93, 1.28)	IGFBP6	0.97 (0.89, 1.05)
IGFBP1	1.01 (0.96, 1.06)	HPX	1.08 (0.92, 1.26)
Treatment^d^	1.28 (0.91, 1.82)	Treatment^b^	1.71 (1.21, 2.42)
Trial^d^	1.00 (0.70, 1.44)	Trial^b^	1.08 (0.76, 1.53)

^a^CHD analyses based on 596 combined cases and controls; stroke analyses based on 620 combined cases and controls.

^b^All analyses include baseline age, prior history of study disease, systolic and diastolic blood pressure, smoking history, treated diabetes history, prior HT use, and body mass index as regression variables to control confounding.

^c^Significant at *P* = 0.05.

^d^Treatment, 1 - active; 0 - placebo; Trial, 1 - E + P trial (no hysterectomy), 0 - E-alone trial (post-hysterectomy).

In Table 5, the year-1 protein concentrations are included in analyses like those in Table 3, but based on cases occurring after year 1 and their disease-specific controls. Estimated ORs are shown for a 30% increment in baseline analyte, and for a 30% increment in the ratio of year 1 to baseline concentration (that is, 30% ‘change’). Toward assessing mediation of HT effects, treatment ORs are shown with only baseline biomarkers included in the analysis, along with corresponding ORs when the analyte change is added to the OR model. For CHD, the treatment OR was essentially unchanged when B2M change was added to the analysis, though there was a suggestion of higher risk (OR of 1.13) among women having a positive concentration change. Interestingly, even though evidence for an association of baseline IGFBP1 with CHD risk was weak, there was a nearly significant association (*P* = 0.06) of risk with change from baseline to year 1 in IGFBP1 concentration, and the OR for treatment was null (OR = 0.96) after controlling for IGFBP1 change.

**Table 5:** **ORs** (**95% CIs) for a 30% increment in baseline biomarker, and in year 1 to baseline biomarker ratio** (**change), based on cases occurring after year 1 and disease-specific controls, from the WHI estrogen plus progestin and estrogen-alone HT trials**

**CHD**			
**Modeled variables**	**N**^**a**^	**Biomarker values included**	
		**Baseline only**	**Baseline + change**
Treatment^a^	491	1.14 (0.79, 1.66)^b^	1.16 (0.80, 1.68)
B2M baseline		1.22 (1.06, 1.40)	1.13 (0.95, 1.34)
B2M change			1.12 (0.96, 1.32)
Trial^a^		1.09 (0.74, 1.59)	1.08 (0.74, 1.59)
Treatment^a^	489	1.21 (0.83, 1.75)^b^	1.24 (0.83, 1.84)
ORM1 baseline		1.00 (0.87, 1.16)	0.98 (0.82, 1.18)
ORM1 change			1.03 (0.87, 1.24)
Trial^a^		1.03 (0.71, 1.51)	1.03 (0.71, 1.51)
Treatment^a^	395	1.11 (0.73, 1.68)^b^	1.11 (0.73, 1.68)
THBS1 baseline		0.95 (0.91, 1.00)	0.95 (0.89, 1.01)
THBS1 change			1.01 (0.95, 1.07)
Trial^a^		0.96 (0.66, 1.39)	0.92 (0.60, 1.41)
Treatment^a^	489	1.19 (0.82, 1.72)^b^	1.26 (0.86 1.85)
CFD baseline		1.09 (0.95, 1.24)	0.93 (0.73 1.19)
CFD change			1.20 (0.94 1.52)
Trial^a^		1.03 (0.73, 1.46)	1.06 (0.72 1.56)
Treatment^a^	479	1.18 (0.81, 1.72)^b^	0.96 (0.62, 1.49)
IGFBP1 baseline		1.01 (0.96, 1.06)	0.97 (0.90, 1.03)
IGFBP1 change			1.07 (0.99, 1.14)
Trial^a^		0.98 (0.67, 1.44)	1.01 (0.69, 1.50)
**Stroke**			
**Modeled variables**	**N**^**a**^	**Biomarker values included**	
		**Baseline only**	**Baseline + change**
Treatment^a^	552	1.84 (1.28, 2.64)^b^	1.87 (1.30, 2.70)
B2M baseline		1.10 (0.97, 1.23)	0.99 (0.86, 1.14)
B2M change			1.24 (1.03, 1.50)
Trial^a^		1.10 (0.76, 1.59)	1.13 (0.78, 1.64)
Treatment^a^	508	1.67 (1.14, 2.44)^b^	1.57 (1.05, 2.33)
IGFBP2 baseline		1.09 (1.00, 1.18)	1.14 (1.00, 1.31)
IGFBP2 change			0.94 (0.84, 1.06)
Trial^a^		1.09 (0.74, 1.60)	1.10 (0.75, 1.61)
Treatment^a^	524	1.77 (1.22, 2.57)^b^	1.74 (1.20, 2.53)
IGFBP4 baseline		1.18 (1.04, 1.34)	1.10 (0.93, 1.30)
IGFBP4 change			1.10 (0.94, 1.28)
Trial^a^		1.08 (0.75, 1.30)	1.10 (0.75, 1.61)
Treatment^a^	474	1.60 (1.08, 2.37)^b^	1.60 (1.08, 2.37)
IGFBP6 baseline		0.99 (0.90, 1.09)	0.99 (0.89, 1.11)
IGFBP6 change			0.99 (0.91, 1.09,)
Trial^a^		1.12 (0.75, 1.66)	1.11 (0.75, 1.66)
Treatment^a^	526	1.74 (1.20, 2.51)^b^	1.74 (1.20, 2.51)
HPX baseline		1.06 (0.88, 1.28)	1.07 (0.84, 1.36)
HPX change			0.99 (0.77, 1.29)
Trial^a^		1.11 (0.76, 1.62)	1.11 (0.76, 1.62)

^a^N, total number of cases plus controls; Treatment, 1 - active; 0 - placebo; Trial, 1 - E + P trial (no hysterectomy), 0 - E-alone trial (post-hysterectomy).

^b^All analyses include baseline age, prior history of study disease, systolic and diastolic blood pressure, smoking history, treated diabetes history, prior HT use, and body mass index as regression variables to control confounding.

For stroke, baseline to 1 year B2M change was positively associated with risk, though the treatment OR was not affected by including B2M change in the analysis. Similarly, OR for treatment was little altered when changes in any of the IGF binding proteins or HPX was added to the analytic model.

Additional analyses (not shown) examined treatment effect mediation by each of these proteins when allowing for technical measurement error in protein assessment, with the blind duplicate data used to estimate the measurement error variance for the (log-transformed) protein concentrations. The treatment ORs were little changed from Table 5 after making this measurement error correction. For example, the treatment effect OR (95% CI), in analyses that include measurement error corrected baseline and 1-year IGFBP1 values, was 0.89 (0.54, 1.45). Other work (also not shown) repeated the Table 5 analyses with cases and controls restricted to women who adhered to their assigned medications during the first year of HT trial participation (at least 80% of pills taken), with little change in HT mediation findings. Further analyses repeated Table 3 excluding women who were being treated for diabetes at baseline (Table 1) with very little change in the ORs for the proteomic markers.

## Discussion

Table 6 presents correlations of each of the proteomic markers with plasma measures of lipids, inflammatory factors, and thrombotic factors, as well as insulin, glucose, white blood cell count, and blood pressure, to facilitate the integration of the associations just described with knowledge about CHD and stroke pathogenesis. These measures were available [18,19] at baseline and 1 year, for all cases and controls, except the 100 stroke case-control pairs that were added late to the case-control study. All variables were log-transformed in calculating these correlations (95% CIs). The correlations shown in Table 6, and ORs for a 30% increment in the proteomic marker from analyses like those shown in Table 3, but with each of the variables on the left side of Table 6 included in the logistic regression model, will be described below for each of the proteomic markers in turn.

**Table 6:** **Pearson correlation** (**bolded) and 95% CIs for baseline log-transformed proteomic markers and baseline log-transformed variables involved in CHD and stroke pathophysiology**

**CHD and stroke risk factors**	**Proteomic marker**								
	**B2M**	**ORM1**	**THBS1**	**CFD**	**IGFBP1**	**IGFBP2**	**IGFBP4**	**IGFBP6**	**HPX**
Lipids									
HDL-C	**-0.16**	**-0.23**	**-0.10**	**-0.25**	**0.22**	**0.52**	**-0.25**	**-0.18**	**-0.21**
	(-0.27, -0.05)	(-0.36, -0.09)	(-0.24, 0.06)	(-0.38, -0.11)	(0.08, 0.36)	(0.37, 0.64)	(-0.41, -0.07)	(-0.36, 0.01)	(-0.38, -0.03)
LDL-C	**-0.04**	**0.04**	**0.09**	**-0.01**	**-0.05**	**-0.15**	**0.01**	**0.02**	**0.14**
	(-0.15, 0.08)	(-0.10, 0.19)	(-0.06, 0.24)	(-0.16, 0.13)	(-0.19, 0.10)	(-0.32, 0.04)	(-0.18, 0.19)	(-0.17, 0.21)	(-0.04, 0.32)
Inflammatory markers									
CRP	**-0.04**	**0.02**	**0.12**	**-0.04**	**0.05**	**-0.11**	**-0.02**	**-0.04**	**0.13**
	(-0.15, 0.08)	(-0.12, 0.17)	(-0.03, 0.27)	(-0.18, 0.11)	(-0.10, 0.19)	(-0.28, 0.08)	(-0.21, 0.16)	(-0.23, 0.15)	(-0.06, 0.30)
IL-6	**0.09**	**0.30**	**0.09**	**0.05**	**-0.11**	**-0.16**	**0.30**	**0.21**	**0.23**
	(-0.03, 0.20)	(0.16, 0.43)	(-0.07, 0.24)	(-0.10, 0.20)	(-0.25, 0.04)	(-0.34, 0.03)	(0.12, 0.46)	(0.01, 0.39)	(0.05, 0.40)
MMP-9	**0.08**	**0.30**	**-0.06**	**0.11**	**-0.06**	**-0.20**	**0.06**	**0.09**	**0.07**
	(-0.03, 0.20)	(0.16, 0.43)	(-0.21, 0.10)	(-0.03, 0.26)	(-0.20, 0.09)	(-0.37, -0.01)	(-0.12, 0.24)	(-0.11, 0.27)	(-0.11, 0.25)
Thrombosis and other blood markers									
Factor VIII	**0.21**	**0.14**	**0.06**	**-0.02**	**0.08**	**-0.11**	**0.23**	**0.19**	**0.12**
	(0.10, 0.32)	(0.00, 0.28)	(-0.10, 0.21)	(-0.16, 0.12)	(-0.07, 0.22)	(-0.29, 0.08)	(0.05, 0.39)	(0.00, 0.37)	(-0.06, 0.30)
Factor 1.2	**0.01**	**-0.04**	**-0.02**	**0.08**	**0.09**	**0.06**	**-0.02**	**0.06**	**0.31**
	(-0.10, 0.13)	(-0.18, 0.10)	(-0.18, 0.13)	(-0.07, 0.22)	(-0.06, 0.23)	(-0.13, 0.24)	(-0.20, 0.16)	(-0.13, 0.25)	(0.14, 0.47)
D-Dimer	**-0.02**	**0.15**	**-0.10**	**0.08**	**0.11**	**-0.04**	**0.00**	**0.00**	**0.15**
	(-0.14, 0.10)	(0.00, 0.29)	(-0.26, 0.06)	(-0.07, 0.23)	(-0.04, 0.26)	(-0.23, 0.15)	(-0.19, 0.18)	(-0.19, 0.20)	(-0.04, 0.32)
vWF	**0.13**	**0.22**	**0.08**	**0.36**	**-0.01**	**0.15**	**0.18**	**0.09**	**0.06**
	(0.02, 0.24)	(0.07, 0.35)	(-0.22, 0.08)	(0.23, 0.48)	(-0.16, 0.13)	(-0.03, 0.33)	(0.00, 0.35)	(-0.10, 0.28)	(-0.13, 0.24)
Insulin	**0.23**	**-0.03**	**-0.01**	**0.19**	**-0.04**	**0.02**	**0.29**	**0.23**	**0.04**
	(0.12, 0.33)	(-0.17, 0.11)	(-0.16, 0.14)	(0.05, 0.32)	(-0.18, 0.11)	(-0.16, 0.21)	(0.11, 0.44)	(0.04, 0.40)	(-0.14, 0.22)
Glucose	**0.03**	**0.17**	**0.02**	**0.24**	**-0.44**	**-0.13**	**0.29**	**0.28**	**0.26**
	(-0.08, 0.15)	(0.02, 0.31)	(-0.14, 0.17)	(0.10, 0.38)	(-0.55, -0.31)	(-0.31, 0.07)	(0.10, 0.45)	(0.08, 0.45)	(0.07, 0.43)
WBC	**0.01**	**0.02**	**0.06**	**0.02**	**-0.10**	**-0.30**	**0.09**	**0.21**	**0.11**
	(-0.10, 0.12)	(-0.12, 0.17)	(-0.09, 0.21)	(-0.12, 0.16)	(-0.24, 0.04)	(-0.46, -0.12)	(-0.10, 0.27)	(0.02, 0.39)	(-0.08, 0.29)
Blood pressure									
Systolic BP	**0.08**	**0.10**	**0.17**	**0.04**	**-0.03**	**-0.25**	**0.00**	**0.17**	**0.14**
	(-0.04, 0.19)	(-0.04, 0.24)	(0.02, 0.32)	(-0.11, 0.18)	(-0.17, 0.12)	(-0.42, -0.07)	(-0.18, 0.18)	(-0.02, 0.35)	(-0.04, 0.31)
Diastolic BP	**0.18**	**0.08**	**-0.11**	**0.19**	**0.00**	**-0.06**	**0.18**	**0.18**	**0.05**
	(0.08, 0.28)	(-0.06, 0.22)	(-0.25, 0.05)	(0.05, 0.32)	(-0.15, 0.14)	(-0.21, 0.09)	(0.03, 0.32)	(0.03, 0.33)	(-0.10, 0.19)

Correlations are based on data from 357 controls drawn from the placebo groups of the WHI postmenopausal HT trials of estrogen plus progestin and estrogen-alone. Estimated correlations are bolded for ease of viewing.

BP: blood pressure; CRP: C-reactive protein; Factor 1.2: prothrombin fragment 1.2; Factor VIII: clotting factor eight; HDL-C: high-density lipoprotein cholesterol; IL-6: interleukin 6; LDL-C: low-density lipoprotein cholesterol; MMP-9: matrix metallaprotinase-9; vWF: von villebrand factor; WBC: total leukocyte count; WHI: Women’s Health Initiative.

This study confirms plasma B2M to be a risk marker for both CHD and stroke, over an average follow-up period of about 4 years for CHD, and longer for stroke. B2M is an amyloidogenic protein that is elevated in hemodialysis patients [26,27], and has been reported to be positively associated with CVD risk factors [28] and with CVD events among patients having chronic kidney disease [29], asymptomatic carotid atherosclerosis [30], or peripheral arterial disease in a healthy elderly population [31]. B2M was found to be increased by about 15% by both types of HT both in the control groups studied here and in our preceding proteomics discovery research [23,24].

From Table 6 it can be seen that B2M has a moderate inverse correlation with HDL-cholesterol, and moderate positive correlations with coagulation factors FVIII and vWF, and with insulin and diastolic blood pressure in this population of postmenopausal women. When the Table 6 variables were added (left side) to the regression model, the OR (95% CI) for a 30% increment in B2M was 1.21 (1.06, 1.37) for CHD, in close proximity to that shown in Table 3 without the addition of these variables, and was 1.46 (1.21, 1.78) for stroke, noticeably stronger than that given in Table 3.

While baseline B2M relates rather clearly to the risk of both CHD and stroke (Table 3), when baseline to 1 year B2M change was added to the regression model (Table 5), it was B2M change that conveyed the greater disease risk, especially for stroke. Recent B2M change deserves consideration as a stroke and possibly a CHD risk marker. However, the HT treatment OR estimates changed little when the B2M change was added to the regression model. Evidently, B2M change is not correlated strongly enough with randomization assignment in the WHI trials for evidence of important mediation of HT effects on these diseases to emerge.

The Table 3 analyses also suggest CFD (adipsin) to be a CHD risk marker, although its association with CHD risk is not significant in analyses that include B2M and the other CHD risk marker candidates (Table 4). CFD correlates inversely with HDL-cholesterol and positively with vWF, insulin, glucose, and diastolic blood pressure. When the Table 6 variables are added to the regression model, the CHD OR (95% CI) for a 30% increment in CFD becomes a non-significant 1.11 (0.96, 1.29). CFD is secreted by adipocytes into the bloodstream. Such adipocytes have been reported to impact multiple functions (blood pressure, lipid metabolism, and hemostasis) to be linked to CVD [32] and to be elevated among obese persons. There is a suggestion (Table 5) that CFD change may relate positively to CHD risk, but the association is not significant.

IGF1 and IGFBP1 have been found to associate positively with all cause and ischemic heart disease mortality in the elderly Rancho Bernardo cohort [33]. Here, baseline IGFBP1 was positively correlated with HDL-cholesterol and inversely associated with glucose. After including these and the other Table 6 measures in the regression analysis, the CHD OR (95% CI) for a 30% IGFBP1 increment was 1.08 (1.02, 1.14). Also, the IGFBP1 change from baseline to year 1 was nearly significant (*P* = 0.06; Table 5) in its association with disease risk. Moreover, the HT treatment OR was reduced to a null value (0.96) after allowing for the IGFBP1 change. Hence, IGFBP1 deserves consideration among biomarkers that may help to explain HT effects on CHD. This mediation possibility may be dampened, however, by the suggestively larger IGFBP1 changes with E-alone *versus* E + P [24], whereas CHD ORs were somewhat larger for E + P [11] than for E-alone [12]. The HT regimes studied here are taken orally, and the first-pass hepatic metabolism is known to stimulate a wide variety of proteins. IGFBP1 is recognized as a liver-selective protein [34], so that this protein may be unaffected by transdermal estrogens, that are being increasingly used in clinical practice to treat menopausal symptoms, since these bypass the liver.

THBS1 has a modest inverse association with CHD risk, consistent with our discovery work [17]. This protein has little correlation with the Table 6 factors, though there is some positive association with systolic blood pressure. The OR (95% CI) for a 30% increment in THBS1 is 0.95 (0.91, 1.00) after including all measures on the left side of Table 6 in the analysis. Thrombin, an important factor in relation to inflammation, coagulation, and wound healing, has been shown to regulate THBS1 expression in endothelial cells [35].

The analyses presented here provide little support for ORM1 as a risk marker for CHD among postmenopausal women. It relates inversely to HDL-cholesterol, and positively to IL-6 and MMP-9, D-dimer, vWF, and glucose. When the Table 6 variables are included in the regression analysis, the OR (95% CI) for a 30% increment in ORM1 is 0.84 (0.77, 1.00), suggesting a possible weak inverse incremental association.

There is an extensive literature, reviewed in [36], on the key role of the insulin-like growth factor system in central nervous system development, function, and repair in animal models. The six IGFBPs coordinate and regulate the biologic activity of IGF1 and IGF2. In spite of sequence homology, the IGFBPs may have quite different biological activity, due to differing abundances, with IGFBP-2, -4, and -5 predominating in the brain, and due to post-translational modifications [36,37]. Also, IGF1 and IGF2 levels have been found to associate inversely with ischemic stroke risk in a Danish case-control study [38], and also with stroke outcome in human studies [39,40].

Here, our validation exercises confirm a positive association of plasma IGFBP2 and IGFBP4 with stroke risk among postmenopausal women. An association was not confirmed for IGFBP6, which has low abundance in the brain in animal models [36,37]. From Table 6 one sees that baseline IGFBP4 and IGFBP6 have quite similar correlation patterns with cardiovascular risk biomarkers, with a negative correlation with HDL-cholesterol and positive correlation with IL-6, MMP-9, Factor VIII, insulin, glucose, and diastolic blood pressure. These patterns are also very similar to those for B2M. In contrast, IGFBP2 has a fairly strong positive correlation with HDL-cholesterol, and negative correlations with MMP-9, WBC, and systolic blood pressure. When the Table 6 variables are included in the analysis, the OR (95% CI) for a 30% increment in IGFBP2 is 1.21 (1.08, 1.35), larger than that given in Table 3, while corresponding ORs (95% CIs) are 1.14 (0.99, 1.32) for IGFBP4 and 1.06 (0.95, 1.17) for IGFBP6. In conjunction with Table 4, one can infer that IGFBP4 and IGFBP2 are associated with stroke risk beyond that attributable to the established biomarkers considered here, while there is little evidence for further association with IGFBP6. However, there was limited evidence of important mediation of HT effects on stroke by either IGFBP4 or IGFBP2, and only a weak suggestion of a positive association between IGFBP4 change from baseline to year 1 and stroke risk.

Hemopexin had been shown in mice to be neuroprotective through high-affinity binding of the pre-oxidant free heme [41]. The baseline HPX measures obtained here correlated negatively with HDL-cholesterol and positively with IL-6, Factor 1.2, and glucose, and the OR (95% CI) for a 30% increment in HPX was 1.05 (0.87, 1.28) after including each of the Table 6 variables in the analysis. Overall, neither baseline nor change in HPX was related to stroke risk in these analyses, but this could be due to the poor reliability of the HPX ELISA assay used here.

The proteins studied here provide some interesting leads concerning the pathophysiology of both CHD and stroke. Their ability to enhance discrimination between cases and controls, however, appears to be limited. For example, when baseline values of these proteins were added to a model that includes the variables used here to control confounding (Table 3 analyses), the AUC for CHD did not increase from its value of 0.670 with 95% CI of (0.615, 0.726) without such addition. For stroke there was some modest AUC increase from 0.645 (0.590, 0.700) without any such proteins added, to 0.663 (0.604, 0.718) when B2M was added, and to 0.665 (0.605, 0.722) when IGFBP4 was added, based on analyses that randomly divided the data into training and validation subsets with the model fitted in the training set used to estimate AUC in the validation set.

Using the same split sample approach, one can estimate positive (PPV) and negative (NPV) predictive values for the proteins for which there is evidence of disease risk association at, say, a specificity of 80%. The PPV and NPV estimates for CHD are 0.70 and 0.58, respectively, without inclusion of the proteins studied here, and are essentially unchanged when any of B2M, CFD, THBS1, or IGFBP1 is added to the model. The corresponding estimated PPV and NPV values of 0.62 and 0.54 without the proteins evaluated increased slightly to 0.68 and 0.58 when B2M was added, to 0.66 and 0.57 when IGFBP4 was added, and to 0.64 and 0.56 when IGFBP2 was added to the regression model. The highly overlapping distribution of the proteomic markers between cases and controls (Table 2) prevents these measures from adding much to case *versus* control discrimination or, presumably, to personalized risk assessment. Even though the discovery and validation phases of this work took place in distinct cohorts, the WHI observational study and clinical trial, respectively, the two cohorts were drawn from essentially the same catchment population, and evaluation of these findings in other populations will be useful.

The set of proteins studied here was limited by our requirement of a commercially available ELISA and not recognized as CHD or stroke risk markers. There were several other proteins within FDR <0.20 bins that could be evaluated, perhaps using multiple reaction monitoring mass spectrometry. These proteins are listed in Tables 1 and 2 of [17].

## Conclusions

Proteomic discovery work has led to the identification of plasma B2M, CFD, and THBS1 as novel risk markers for CHD. Additionally, an increase in IGFBP1 over a 1-year period also appears to convey CHD risk, and may be relevant to the early elevation in CHD risk among women initiating oral HT. This work has also led to the identification of plasma B2M, IGFBP2, and especially IGFBP4 as novel risk markers for stroke risk among postmenopausal women.

## Abbreviations

AUC: Area under receiver-operator curve; B2M: Beta-2-microglobulin; CFD: Complement factor D pre-protein; CHD: Coronary heart disease; CI: Confidence interval; CRP: C-reactive protein; CVD: Cardiovascular disease; E + P: Estrogen plus progestin; E-alone: Estrogen-alone; ELISA: Enzyme-linked immunosorbent assay; Factor 1.2: Prothrombin fragment 1.2; Factor VIII: Clotting factor eight; FDR: False discovery rate; HDL-C: High-density lipoprotein cholesterol; HPX: Hemopexin; HT: (postmenopausal) hormone therapy; IGFBP: Insulin-like growth factor binding protein; IL-6: Interleukin 6; LDL-C: Low-density lipoprotein cholesterol; MMP-9: Matrix metallaprotinase-9; OR: Odds ratio; ORM1: Alpha-1-acid glycoprotein 1; THBS1: Thrombospondin-1; vWF: Von villebrand factor; WBC: White blood cell count; WHI: Women’s Health Initiative.

## Competing interests

The authors declare that they have no competing interests.

## Authors’ contributions

RLP and SMH secured funding, oversaw the data collection, and drafted the manuscript. MJ conducted the ELISA assays and related quality assurance activities. AA assembled project data and provided ongoing data analyses support. SZ was the principal data analyst for manuscript development, and shared in the initial drafting of the manuscript. JH, RDJ, JER, and JEM served as consultants throughout the project, providing cardiovascular disease expertise and input to analyte selection. All authors provided review and critical input to manuscript development and finalization. All authors read and approved the final manuscript.
